# 2-Phenyl-5,6,7,8-tetra­hydro­imidazo[2,1-*b*][1,3]benzo­thia­zole

**DOI:** 10.1107/S1600536814010885

**Published:** 2014-05-17

**Authors:** Alexander S. Bunev, Elena V. Sukhonosova, Petr P. Purygin, Gennady I. Ostapenko, Victor N. Khrustalev

**Affiliations:** a Department of Chemistry and Chemical Technology, Togliatti State University, 14 Belorusskaya St, Togliatti 445667, Russian Federation; b Department of Organic, Bioorganic and Medicinal Chemistry, Samara State University, 1 Akademician Pavlov St, Samara 443011, Russian Federation; c X-Ray Structural Centre, A.N. Nesmeyanov Institute of Organoelement Compounds, Russian Academy of Sciences, 28 Vavilov Street, B–334, Moscow 119991, Russian Federation

## Abstract

The title compound, C_15_H_14_N_2_S, crystallizes with two independent mol­ecules in the asymmetric unit. The central imidazo[2,1-*b*][1,3]benzo­thia­zole unit is planar (r.m.s. deviations of 0.010 and 0.008 Å for the two independent mol­ecules). The fused tetra­hydro­hexane ring adopts a half-chair conformation. The phenyl substituent is twisted by 16.96 (13) and 22.89 (12)° relative to the central imidazo[2,1-*b*][1,3]benzo­thia­zole unit in the two mol­ecules. In the crystal, there are no significant intermolecular interactions present.

## Related literature   

For applications of imidazo[2,1-*b*][1,3]benzo­thia­zoles, see: Ager *et al.* (1988 ▶); Sanfilippo *et al.* (1988 ▶); Barchéchath *et al.* (2005 ▶); Andreani *et al.* (2008 ▶); Chao *et al.* (2009 ▶); Kumbhare *et al.* (2011 ▶); Chandak *et al.* (2013 ▶). For the crystal structures of related compounds, see: Landreau *et al.* (2002 ▶); Adib *et al.* (2008 ▶); Fun, Asik *et al.* (2011 ▶); Fun, Hemamalini *et al.* (2011 ▶); Ghabbour *et al.* (2012 ▶); Bunev *et al.* (2013*a* ▶,*b* ▶, 2014 ▶).
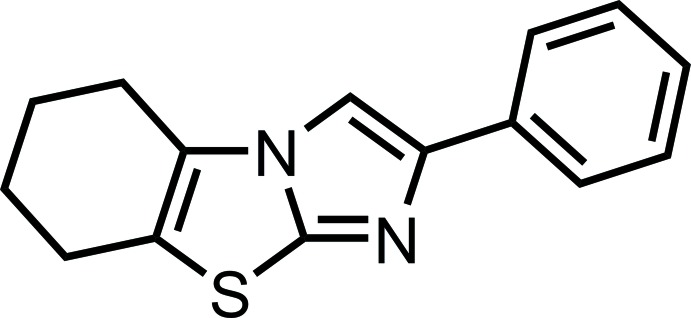


## Experimental   

### 

#### Crystal data  

C_15_H_14_N_2_S
*M* *_r_* = 254.34
Monoclinic, 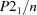

*a* = 12.523 (3) Å
*b* = 10.699 (3) Å
*c* = 18.930 (5) Å
β = 102.291 (6)°
*V* = 2478.2 (11) Å^3^
*Z* = 8
Mo *K*α radiation
μ = 0.24 mm^−1^
*T* = 120 K
0.30 × 0.05 × 0.03 mm

#### Data collection  

Bruker APEXII CCD diffractometer
Absorption correction: multi-scan (*SADABS*; Bruker, 2003 ▶) *T*_min_ = 0.931, *T*_max_ = 0.993
33479 measured reflections
7558 independent reflections
3223 reflections with *I* > 2σ(*I*)
*R*_int_ = 0.068

#### Refinement  

*R*[*F* ^2^ > 2σ(*F* ^2^)] = 0.065
*wR*(*F* ^2^) = 0.157
*S* = 0.93
7558 reflections
325 parameters
H-atom parameters constrained
Δρ_max_ = 0.36 e Å^−3^
Δρ_min_ = −0.45 e Å^−3^

### 

Data collection: *APEX2* (Bruker, 2005 ▶); cell refinement: *SAINT* (Bruker, 2001 ▶); data reduction: *SAINT*; program(s) used to solve structure: *SHELXTL* (Sheldrick, 2008 ▶); program(s) used to refine structure: *SHELXTL*; molecular graphics: *SHELXTL*; software used to prepare material for publication: *SHELXTL*.

## Supplementary Material

Crystal structure: contains datablock(s) global, I. DOI: 10.1107/S1600536814010885/rk2428sup1.cif

Structure factors: contains datablock(s) I. DOI: 10.1107/S1600536814010885/rk2428Isup2.hkl

Supporting information file. DOI: 10.1107/S1600536814010885/rk2428Isup3.cml

CCDC reference: 1002491

Additional supporting information:  crystallographic information; 3D view; checkCIF report

## Figures and Tables

**Fig. 1.**
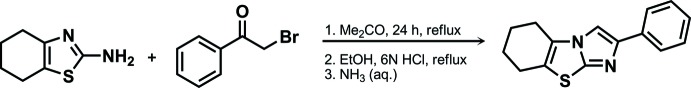
Synthesis of 2-phenyl-5,6,7,8-tetrahydroimidazo[2,1–b][1,3]benzothiazole.

**Fig. 2.**
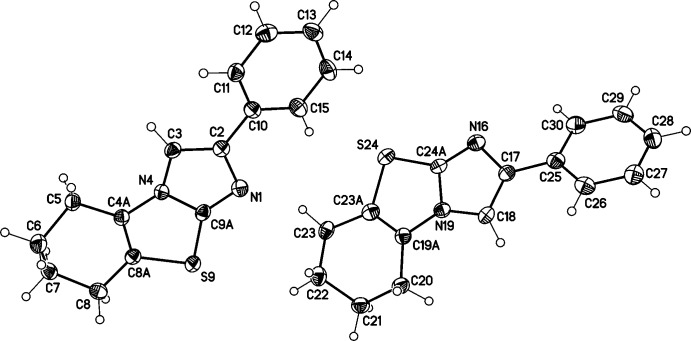
Molecular structure of I (two crystallographically independent molecules are presented). Displacement ellipsoids are shown at the 50% probability level. H atoms are depicted as small spheres of arbitrary radius.

## supplementary crystallographic information

### 1. Comment

Imidazo[2,1-*b*][1,3]benzothiazole are of great interest due to their biological properties. These compounds and their derivatives demonstrate the antitumor (Andreani *et al.*, 2008), antiallergic (Ager *et al.*, 1988), anesthetic (Sanfilippo *et al.*, 1988) and anti-cancer (Kumbhare *et al.*, 2011) activities as well as the inhibition activity of apoptosis in testiculargerm cells (Chandak *et al.*, 2013), lymphocytes (Barchéchath *et al.*, 2005), and FMS-like tyrosine kinase-3 (FLT3) (Chao *et al.*, 2009).

In this work, a 5,6,7,8-tetrahydroimidazo[2,1-*b*][1,3]benzothiazole, C_15_H_14_N_2_S, (I) was prepared by the reaction of 5,6,7,8-tetrahydrobenzothiazole-2-amine with 2-bromo-1-phenylethanone (Fig. 1), and its structure was unambiguously established by the X-ray diffraction study (Fig. 2).

The bond lengths and angles within the molecule of I are in a good agreement with those found in the related compounds (Landreau *et al.*, 2002; Adib *et al.*, 2008; Fun, Asik *et al.*, 2011; Fun, Hemamalini *et al.*, 2011; Ghabbour *et al.*, 2012; Bunev *et al.*, 2013*a*,*b*; Bunev *et al.*, 2014).

The title compound, C_15_H_14_N_2_S (I), crystallizes with two crystallographically independent molecules in the asymmetric unit (Fig. 2). The central imidazo[2,1-*b*][1,3]benzothiazole moiety is planar (r.m.s. deviation = 0.010 and 0.008 Å, respectively, for the two crystallographically independent molecules). The fused tetrahydrohexane ring adopts a *half-chair* conformation (the C6, C7 and C21, C22 carbon atoms are out of the planes passed through the other atoms of the rings by 0.411 (6), -0.314 (6) and 0.387 (6), -0.355 (6) Å, respectively, for the two crystallographically independent molecules). The phenyl substituent is twisted by 16.96 (13) and 22.89 (12) ° (for the two crystallographically independent molecules, respectively) relative to the central imidazo[2,1-*b*][1,3]benzothiazole moiety.

In the crystal, the molecules of I are arrangement at van der Waals distances.

### 2. Experimental

A mixture of 5,6,7,8-tetrahydrobenzothiazole–2–amine (1.54 g, 10 mmol) and 2–bromo–1–phenylethanone (1.99 g, 10 mmol) was dissolved in acetone (35 mL). The reaction mixture was stirred for 24 h. The resulting precipitate was collected, suspended in *Et*OH (50 mL) containing 6*N* HCl (5 mL) and heated under reflux. After cooling up to room temperature, the solution basified with 20% NH_4_OH yielded the expected 5,6,7,8–tetrahydroimidazo[2,1–*b*][1,3]benzothiazole. The crude product was crystallized from EtOH. Yield is 82%. The single crystals of the product were obtained by slow crystallization from *Et*OH. M.p. = 440–442 K. IR (KBr), ν/cm^-1^: 3137, 2933, 1602, 1539, 1465, 1438, 774, 718, 555. ^1^H NMR (400 MHz, DMSO–*d_6_*, 304 K): δ = 1.74–1.70 (m, 4H), 2.36–2.33 (m, 2H), 2.57–2.54 (m, 2H), 5.76 (s, 1H), 7.64 (t, 2H, *J* = 7.6), 7.78–7.75 (m, 1H), 8.06 (d, 2H, *J* = 7.9). Anal. Calcd for C_15_H_14_N_2_S: C, 70.83; H, 5.55. Found: C, 70.91; H, 5.62.

### 3. Refinement

All hydrogen atoms were placed in the calculated positions with C—H = 0.95 (aryl H) and 0.99 (methylene H) Å and refined in the riding model with fixed isotropic displacement parameters: *U*_iso_(H) = 1.2*U*_eq_(C).

### Crystal data

**Table:**

C_15_H_14_N_2_S	*F*(000) = 1072
*M**_r_* = 254.34	*D*_x_ = 1.363 Mg m^−^^3^
Monoclinic, *P*2_1_/*n*	Melting point = 440–442 K
Hall symbol: -P 2yn	Mo *K*α radiation, λ = 0.71073 Å
*a* = 12.523 (3) Å	Cell parameters from 769 reflections
*b* = 10.699 (3) Å	θ = 2.2–19.4°
*c* = 18.930 (5) Å	µ = 0.24 mm^−^^1^
β = 102.291 (6)°	*T* = 120 K
*V* = 2478.2 (11) Å^3^	Needle, colourless
*Z* = 8	0.30 × 0.05 × 0.03 mm

### Data collection

**Table:**

Bruker APEXII CCD diffractometer	7558 independent reflections
Radiation source: fine-focus sealed tube	3223 reflections with *I* > 2σ(*I*)
Graphite monochromator	*R*_int_ = 0.068
φ and ω scans	θ_max_ = 30.5°, θ_min_ = 1.8°
Absorption correction: multi-scan (*SADABS*; Bruker, 2003)	*h* = −17→17
*T*_min_ = 0.931, *T*_max_ = 0.993	*k* = −15→15
33479 measured reflections	*l* = −27→27

### Refinement

**Table:**

Refinement on *F*^2^	Primary atom site location: structure-invariant direct methods
Least-squares matrix: full	Secondary atom site location: difference Fourier map
*R*[*F*^2^ > 2σ(*F*^2^)] = 0.065	Hydrogen site location: inferred from neighbouring sites
*wR*(*F*^2^) = 0.157	H-atom parameters constrained
*S* = 0.93	*w* = 1/[σ^2^(*F*_o_^2^) + (0.0349*P*)^2^] where *P* = (*F*_o_^2^ + 2*F*_c_^2^)/3
7558 reflections	(Δ/σ)_max_ < 0.001
325 parameters	Δρ_max_ = 0.36 e Å^−^^3^
0 restraints	Δρ_min_ = −0.45 e Å^−^^3^

### Special details

**Table:**

Geometry. All s.u.'s (except the s.u. in the dihedral angle between two l.s. planes) are estimated using the full covariance matrix. The cell s.u.'s are taken into account individually in the estimation of s.u.'s in distances, angles and torsion angles; correlations between s.u.'s in cell parameters are only used when they are defined by crystal symmetry. An approximate (isotropic) treatment of cell s.u.'s is used for estimating s.u.'s involving l.s. planes.
Refinement. Refinement of *F*^2^ against ALL reflections. The weighted *R*–factor w*R* and goodness of fit *S* are based on *F*^2^, conventional *R*–factors *R* are based on *F*, with *F* set to zero for negative *F*^2^. The threshold expression of *F*^2^ > 2σ(*F*^2^) is used only for calculating *R*–factors(gt) etc. and is not relevant to the choice of reflections for refinement. *R*–factors based on *F*^2^ are statistically about twice as large as those based on *F*., and *R*–factors based on ALL data will be even larger.

### Fractional atomic coordinates and isotropic or equivalent isotropic displacement parameters (Å^2^)

**Table:**

	*x*	*y*	*z*	*U*_iso_*/*U*_eq_	
N1	0.3882 (2)	0.6184 (2)	0.11553 (14)	0.0237 (6)	
C2	0.3823 (3)	0.5036 (3)	0.14946 (16)	0.0212 (7)	
C3	0.4704 (3)	0.4301 (3)	0.14592 (16)	0.0219 (7)	
H3	0.4850	0.3481	0.1647	0.026*	
N4	0.5337 (2)	0.5003 (2)	0.10923 (14)	0.0205 (6)	
C4A	0.6340 (3)	0.4902 (3)	0.08809 (16)	0.0209 (7)	
C5	0.7062 (2)	0.3795 (3)	0.10652 (18)	0.0248 (7)	
H5A	0.6796	0.3105	0.0725	0.030*	
H5B	0.7049	0.3508	0.1560	0.030*	
C6	0.8230 (3)	0.4141 (3)	0.10196 (18)	0.0277 (8)	
H6A	0.8567	0.4646	0.1447	0.033*	
H6B	0.8668	0.3370	0.1024	0.033*	
C7	0.8246 (3)	0.4884 (3)	0.03323 (17)	0.0254 (7)	
H7A	0.7920	0.4368	−0.0093	0.030*	
H7B	0.9014	0.5058	0.0309	0.030*	
C8	0.7619 (3)	0.6131 (3)	0.02925 (17)	0.0255 (7)	
H8A	0.8072	0.6760	0.0605	0.031*	
H8B	0.7463	0.6448	−0.0210	0.031*	
C8A	0.6568 (2)	0.5938 (3)	0.05377 (16)	0.0212 (7)	
S9	0.55424 (7)	0.70942 (8)	0.04714 (5)	0.0253 (2)	
C9A	0.4804 (3)	0.6118 (3)	0.09283 (16)	0.0213 (7)	
C10	0.2878 (3)	0.4714 (3)	0.18064 (17)	0.0229 (7)	
C11	0.2908 (3)	0.3708 (3)	0.22804 (17)	0.0250 (7)	
H11	0.3563	0.3241	0.2426	0.030*	
C12	0.1987 (3)	0.3383 (3)	0.25409 (17)	0.0292 (8)	
H12	0.2018	0.2695	0.2862	0.035*	
C13	0.1024 (3)	0.4053 (3)	0.23360 (18)	0.0297 (8)	
H13	0.0393	0.3822	0.2509	0.036*	
C14	0.0992 (3)	0.5066 (3)	0.18754 (18)	0.0297 (8)	
H14	0.0339	0.5539	0.1737	0.036*	
C15	0.1911 (3)	0.5393 (3)	0.16149 (17)	0.0267 (8)	
H15	0.1879	0.6091	0.1301	0.032*	
N16	−0.1213 (2)	0.9124 (2)	0.11849 (14)	0.0252 (6)	
C17	−0.1096 (3)	1.0225 (3)	0.15894 (17)	0.0224 (7)	
C18	−0.0110 (3)	1.0785 (3)	0.15889 (16)	0.0230 (7)	
H18	0.0156	1.1548	0.1818	0.028*	
N19	0.0414 (2)	1.0016 (2)	0.11889 (14)	0.0218 (6)	
C19A	0.1393 (3)	0.9974 (3)	0.09439 (17)	0.0230 (7)	
C20	0.2241 (3)	1.0980 (3)	0.11148 (17)	0.0260 (8)	
H20A	0.2640	1.0906	0.1624	0.031*	
H20B	0.1887	1.1811	0.1049	0.031*	
C21	0.3034 (3)	1.0841 (3)	0.06097 (18)	0.0278 (8)	
H21A	0.3690	1.1358	0.0793	0.033*	
H21B	0.2683	1.1156	0.0124	0.033*	
C22	0.3384 (3)	0.9486 (3)	0.05454 (18)	0.0282 (8)	
H22A	0.3731	0.9166	0.1031	0.034*	
H22B	0.3934	0.9450	0.0239	0.034*	
C23	0.2406 (3)	0.8651 (3)	0.02141 (17)	0.0252 (8)	
H23A	0.2206	0.8784	−0.0315	0.030*	
H23B	0.2608	0.7761	0.0304	0.030*	
C23A	0.1451 (3)	0.8957 (3)	0.05424 (17)	0.0252 (7)	
S24	0.02778 (7)	0.79992 (8)	0.04362 (5)	0.0269 (2)	
C24A	−0.0288 (3)	0.9037 (3)	0.09640 (16)	0.0218 (7)	
C25	−0.1985 (3)	1.0704 (3)	0.19086 (17)	0.0247 (7)	
C26	−0.1767 (3)	1.1565 (3)	0.24797 (17)	0.0266 (8)	
H26	−0.1036	1.1819	0.2669	0.032*	
C27	−0.2608 (3)	1.2052 (3)	0.27725 (17)	0.0284 (8)	
H27	−0.2448	1.2638	0.3158	0.034*	
C28	−0.3676 (3)	1.1686 (3)	0.25042 (18)	0.0309 (8)	
H28	−0.4250	1.2023	0.2704	0.037*	
C29	−0.3911 (3)	1.0825 (3)	0.19427 (19)	0.0320 (8)	
H29	−0.4645	1.0571	0.1760	0.038*	
C30	−0.3071 (3)	1.0334 (3)	0.16478 (18)	0.0288 (8)	
H30	−0.3236	0.9742	0.1266	0.035*	

### Atomic displacement parameters (Å^2^)

**Table:**

	*U*^11^	*U*^22^	*U*^33^	*U*^12^	*U*^13^	*U*^23^
N1	0.0236 (16)	0.0247 (16)	0.0223 (15)	0.0008 (12)	0.0038 (12)	0.0008 (11)
C2	0.0226 (18)	0.0226 (18)	0.0176 (16)	−0.0013 (13)	0.0027 (13)	−0.0033 (13)
C3	0.0264 (19)	0.0189 (17)	0.0217 (17)	0.0003 (13)	0.0082 (14)	0.0012 (13)
N4	0.0203 (15)	0.0194 (14)	0.0222 (15)	0.0007 (11)	0.0056 (11)	−0.0013 (11)
C4A	0.0207 (18)	0.0205 (17)	0.0207 (17)	0.0004 (13)	0.0025 (13)	−0.0032 (13)
C5	0.0242 (19)	0.0234 (18)	0.0280 (19)	0.0038 (14)	0.0087 (14)	0.0019 (14)
C6	0.0255 (19)	0.0277 (19)	0.0293 (19)	0.0043 (15)	0.0041 (15)	0.0022 (15)
C7	0.0232 (18)	0.0280 (19)	0.0267 (19)	−0.0010 (14)	0.0093 (14)	−0.0037 (14)
C8	0.0258 (19)	0.0275 (19)	0.0237 (18)	−0.0024 (14)	0.0065 (14)	0.0008 (14)
C8A	0.0233 (18)	0.0203 (17)	0.0194 (17)	−0.0003 (13)	0.0031 (13)	−0.0019 (13)
S9	0.0254 (5)	0.0210 (4)	0.0300 (5)	0.0024 (4)	0.0073 (4)	0.0033 (4)
C9A	0.0233 (18)	0.0189 (17)	0.0208 (17)	0.0013 (13)	0.0025 (13)	−0.0038 (13)
C10	0.0221 (18)	0.0250 (18)	0.0218 (17)	−0.0025 (13)	0.0053 (14)	−0.0044 (13)
C11	0.0261 (19)	0.0234 (18)	0.0262 (19)	0.0019 (14)	0.0071 (14)	−0.0031 (14)
C12	0.038 (2)	0.0266 (19)	0.0237 (19)	−0.0050 (15)	0.0087 (16)	−0.0005 (14)
C13	0.027 (2)	0.033 (2)	0.031 (2)	−0.0085 (16)	0.0101 (15)	−0.0087 (16)
C14	0.0192 (18)	0.040 (2)	0.029 (2)	0.0017 (15)	0.0030 (15)	−0.0046 (16)
C15	0.0261 (19)	0.033 (2)	0.0224 (18)	−0.0005 (15)	0.0074 (14)	−0.0006 (14)
N16	0.0287 (17)	0.0234 (15)	0.0229 (15)	−0.0014 (12)	0.0040 (12)	0.0014 (12)
C17	0.0249 (19)	0.0205 (17)	0.0209 (17)	0.0003 (13)	0.0032 (14)	0.0006 (13)
C18	0.0278 (19)	0.0188 (17)	0.0220 (18)	0.0022 (14)	0.0038 (14)	−0.0012 (13)
N19	0.0238 (15)	0.0209 (14)	0.0201 (15)	−0.0018 (11)	0.0035 (11)	−0.0012 (11)
C19A	0.0231 (18)	0.0224 (18)	0.0224 (18)	0.0016 (14)	0.0026 (14)	0.0025 (13)
C20	0.0263 (19)	0.0229 (18)	0.0274 (19)	−0.0010 (14)	0.0024 (14)	−0.0018 (14)
C21	0.027 (2)	0.0255 (19)	0.030 (2)	−0.0035 (15)	0.0035 (15)	−0.0016 (15)
C22	0.030 (2)	0.029 (2)	0.0267 (19)	0.0067 (15)	0.0085 (15)	0.0044 (14)
C23	0.031 (2)	0.0228 (18)	0.0215 (18)	0.0062 (14)	0.0054 (15)	0.0026 (13)
C23A	0.029 (2)	0.0241 (19)	0.0217 (18)	0.0014 (14)	0.0037 (14)	0.0059 (14)
S24	0.0317 (5)	0.0198 (4)	0.0286 (5)	−0.0002 (4)	0.0051 (4)	−0.0023 (4)
C24A	0.0260 (19)	0.0176 (17)	0.0213 (17)	−0.0016 (13)	0.0037 (14)	0.0000 (13)
C25	0.028 (2)	0.0225 (18)	0.0238 (18)	0.0006 (14)	0.0050 (14)	0.0070 (14)
C26	0.027 (2)	0.030 (2)	0.0217 (18)	−0.0011 (14)	0.0031 (14)	0.0086 (14)
C27	0.036 (2)	0.0271 (19)	0.0227 (18)	0.0041 (16)	0.0075 (15)	0.0067 (15)
C28	0.033 (2)	0.031 (2)	0.032 (2)	0.0081 (16)	0.0136 (16)	0.0109 (15)
C29	0.024 (2)	0.032 (2)	0.039 (2)	−0.0034 (15)	0.0054 (16)	0.0086 (17)
C30	0.029 (2)	0.030 (2)	0.0269 (19)	−0.0025 (15)	0.0032 (15)	0.0002 (14)

### Geometric parameters (Å, º)

**Table:**

N1—C9A	1.317 (4)	N16—C24A	1.316 (4)
N1—C2	1.395 (4)	N16—C17	1.395 (4)
C2—C3	1.368 (4)	C17—C18	1.373 (4)
C2—C10	1.472 (4)	C17—C25	1.468 (4)
C3—N4	1.382 (4)	C18—N19	1.376 (4)
C3—H3	0.9500	C18—H18	0.9500
N4—C9A	1.370 (4)	N19—C24A	1.376 (4)
N4—C4A	1.400 (4)	N19—C19A	1.400 (4)
C4A—C8A	1.346 (4)	C19A—C23A	1.338 (4)
C4A—C5	1.486 (4)	C19A—C20	1.498 (4)
C5—C6	1.529 (4)	C20—C21	1.526 (4)
C5—H5A	0.9900	C20—H20A	0.9900
C5—H5B	0.9900	C20—H20B	0.9900
C6—C7	1.528 (4)	C21—C22	1.527 (4)
C6—H6A	0.9900	C21—H21A	0.9900
C6—H6B	0.9900	C21—H21B	0.9900
C7—C8	1.542 (4)	C22—C23	1.538 (4)
C7—H7A	0.9900	C22—H22A	0.9900
C7—H7B	0.9900	C22—H22B	0.9900
C8—C8A	1.500 (4)	C23—C23A	1.497 (4)
C8—H8A	0.9900	C23—H23A	0.9900
C8—H8B	0.9900	C23—H23B	0.9900
C8A—S9	1.769 (3)	C23A—S24	1.767 (3)
S9—C9A	1.742 (3)	S24—C24A	1.742 (3)
C10—C15	1.392 (4)	C25—C30	1.401 (4)
C10—C11	1.397 (4)	C25—C26	1.402 (4)
C11—C12	1.392 (4)	C26—C27	1.392 (4)
C11—H11	0.9500	C26—H26	0.9500
C12—C13	1.385 (5)	C27—C28	1.382 (5)
C12—H12	0.9500	C27—H27	0.9500
C13—C14	1.386 (5)	C28—C29	1.390 (5)
C13—H13	0.9500	C28—H28	0.9500
C14—C15	1.390 (4)	C29—C30	1.395 (5)
C14—H14	0.9500	C29—H29	0.9500
C15—H15	0.9500	C30—H30	0.9500
			
C9A—N1—C2	103.8 (3)	C24A—N16—C17	103.7 (3)
C3—C2—N1	111.2 (3)	C18—C17—N16	111.0 (3)
C3—C2—C10	127.7 (3)	C18—C17—C25	127.6 (3)
N1—C2—C10	121.1 (3)	N16—C17—C25	121.3 (3)
C2—C3—N4	105.5 (3)	C17—C18—N19	105.9 (3)
C2—C3—H3	127.2	C17—C18—H18	127.1
N4—C3—H3	127.2	N19—C18—H18	127.1
C9A—N4—C3	106.4 (3)	C18—N19—C24A	106.1 (3)
C9A—N4—C4A	115.2 (3)	C18—N19—C19A	139.1 (3)
C3—N4—C4A	138.3 (3)	C24A—N19—C19A	114.8 (3)
C8A—C4A—N4	111.7 (3)	C23A—C19A—N19	111.8 (3)
C8A—C4A—C5	126.0 (3)	C23A—C19A—C20	125.8 (3)
N4—C4A—C5	122.2 (3)	N19—C19A—C20	122.4 (3)
C4A—C5—C6	109.6 (3)	C19A—C20—C21	108.7 (3)
C4A—C5—H5A	109.7	C19A—C20—H20A	109.9
C6—C5—H5A	109.7	C21—C20—H20A	109.9
C4A—C5—H5B	109.7	C19A—C20—H20B	109.9
C6—C5—H5B	109.7	C21—C20—H20B	109.9
H5A—C5—H5B	108.2	H20A—C20—H20B	108.3
C7—C6—C5	111.1 (3)	C20—C21—C22	112.2 (3)
C7—C6—H6A	109.4	C20—C21—H21A	109.2
C5—C6—H6A	109.4	C22—C21—H21A	109.2
C7—C6—H6B	109.4	C20—C21—H21B	109.2
C5—C6—H6B	109.4	C22—C21—H21B	109.2
H6A—C6—H6B	108.0	H21A—C21—H21B	107.9
C6—C7—C8	113.1 (3)	C21—C22—C23	111.5 (3)
C6—C7—H7A	109.0	C21—C22—H22A	109.3
C8—C7—H7A	109.0	C23—C22—H22A	109.3
C6—C7—H7B	109.0	C21—C22—H22B	109.3
C8—C7—H7B	109.0	C23—C22—H22B	109.3
H7A—C7—H7B	107.8	H22A—C22—H22B	108.0
C8A—C8—C7	109.7 (3)	C23A—C23—C22	109.9 (3)
C8A—C8—H8A	109.7	C23A—C23—H23A	109.7
C7—C8—H8A	109.7	C22—C23—H23A	109.7
C8A—C8—H8B	109.7	C23A—C23—H23B	109.7
C7—C8—H8B	109.7	C22—C23—H23B	109.7
H8A—C8—H8B	108.2	H23A—C23—H23B	108.2
C4A—C8A—C8	123.6 (3)	C19A—C23A—C23	124.0 (3)
C4A—C8A—S9	112.6 (2)	C19A—C23A—S24	113.1 (3)
C8—C8A—S9	123.6 (2)	C23—C23A—S24	123.0 (2)
C9A—S9—C8A	89.97 (15)	C24A—S24—C23A	89.70 (15)
N1—C9A—N4	113.1 (3)	N16—C24A—N19	113.3 (3)
N1—C9A—S9	136.4 (3)	N16—C24A—S24	136.1 (3)
N4—C9A—S9	110.5 (2)	N19—C24A—S24	110.6 (2)
C15—C10—C11	118.2 (3)	C30—C25—C26	118.2 (3)
C15—C10—C2	120.2 (3)	C30—C25—C17	121.4 (3)
C11—C10—C2	121.6 (3)	C26—C25—C17	120.4 (3)
C12—C11—C10	120.6 (3)	C27—C26—C25	120.9 (3)
C12—C11—H11	119.7	C27—C26—H26	119.6
C10—C11—H11	119.7	C25—C26—H26	119.6
C13—C12—C11	120.6 (3)	C28—C27—C26	120.2 (3)
C13—C12—H12	119.7	C28—C27—H27	119.9
C11—C12—H12	119.7	C26—C27—H27	119.9
C12—C13—C14	119.2 (3)	C27—C28—C29	120.0 (3)
C12—C13—H13	120.4	C27—C28—H28	120.0
C14—C13—H13	120.4	C29—C28—H28	120.0
C13—C14—C15	120.4 (3)	C28—C29—C30	120.1 (3)
C13—C14—H14	119.8	C28—C29—H29	120.0
C15—C14—H14	119.8	C30—C29—H29	120.0
C14—C15—C10	121.1 (3)	C29—C30—C25	120.7 (3)
C14—C15—H15	119.5	C29—C30—H30	119.6
C10—C15—H15	119.5	C25—C30—H30	119.6
			
C9A—N1—C2—C3	−0.5 (3)	C24A—N16—C17—C18	1.2 (4)
C9A—N1—C2—C10	−177.9 (3)	C24A—N16—C17—C25	177.0 (3)
N1—C2—C3—N4	0.3 (4)	N16—C17—C18—N19	−1.1 (4)
C10—C2—C3—N4	177.5 (3)	C25—C17—C18—N19	−176.5 (3)
C2—C3—N4—C9A	0.0 (3)	C17—C18—N19—C24A	0.5 (3)
C2—C3—N4—C4A	177.0 (3)	C17—C18—N19—C19A	178.2 (3)
C9A—N4—C4A—C8A	−1.3 (4)	C18—N19—C19A—C23A	−178.6 (3)
C3—N4—C4A—C8A	−178.1 (3)	C24A—N19—C19A—C23A	−1.0 (4)
C9A—N4—C4A—C5	175.3 (3)	C18—N19—C19A—C20	0.5 (6)
C3—N4—C4A—C5	−1.5 (6)	C24A—N19—C19A—C20	178.1 (3)
C8A—C4A—C5—C6	17.0 (4)	C23A—C19A—C20—C21	14.3 (4)
N4—C4A—C5—C6	−159.1 (3)	N19—C19A—C20—C21	−164.7 (3)
C4A—C5—C6—C7	−45.4 (4)	C19A—C20—C21—C22	−45.1 (4)
C5—C6—C7—C8	61.3 (4)	C20—C21—C22—C23	62.7 (4)
C6—C7—C8—C8A	−42.5 (4)	C21—C22—C23—C23A	−43.5 (4)
N4—C4A—C8A—C8	175.9 (3)	N19—C19A—C23A—C23	−179.2 (3)
C5—C4A—C8A—C8	−0.6 (5)	C20—C19A—C23A—C23	1.7 (5)
N4—C4A—C8A—S9	0.6 (3)	N19—C19A—C23A—S24	0.8 (4)
C5—C4A—C8A—S9	−175.9 (3)	C20—C19A—C23A—S24	−178.3 (2)
C7—C8—C8A—C4A	12.9 (4)	C22—C23—C23A—C19A	13.0 (4)
C7—C8—C8A—S9	−172.2 (2)	C22—C23—C23A—S24	−167.0 (2)
C4A—C8A—S9—C9A	0.2 (3)	C19A—C23A—S24—C24A	−0.3 (3)
C8—C8A—S9—C9A	−175.1 (3)	C23—C23A—S24—C24A	179.7 (3)
C2—N1—C9A—N4	0.6 (3)	C17—N16—C24A—N19	−0.9 (3)
C2—N1—C9A—S9	−178.9 (3)	C17—N16—C24A—S24	−179.3 (3)
C3—N4—C9A—N1	−0.4 (4)	C18—N19—C24A—N16	0.3 (4)
C4A—N4—C9A—N1	−178.2 (3)	C19A—N19—C24A—N16	−178.0 (3)
C3—N4—C9A—S9	179.2 (2)	C18—N19—C24A—S24	179.1 (2)
C4A—N4—C9A—S9	1.5 (3)	C19A—N19—C24A—S24	0.8 (3)
C8A—S9—C9A—N1	178.6 (4)	C23A—S24—C24A—N16	178.2 (4)
C8A—S9—C9A—N4	−0.9 (2)	C23A—S24—C24A—N19	−0.3 (2)
C3—C2—C10—C15	−160.7 (3)	C18—C17—C25—C30	154.6 (3)
N1—C2—C10—C15	16.3 (5)	N16—C17—C25—C30	−20.4 (5)
C3—C2—C10—C11	17.3 (5)	C18—C17—C25—C26	−24.4 (5)
N1—C2—C10—C11	−165.7 (3)	N16—C17—C25—C26	160.6 (3)
C15—C10—C11—C12	1.2 (5)	C30—C25—C26—C27	−0.9 (5)
C2—C10—C11—C12	−176.8 (3)	C17—C25—C26—C27	178.1 (3)
C10—C11—C12—C13	−0.1 (5)	C25—C26—C27—C28	0.3 (5)
C11—C12—C13—C14	−0.9 (5)	C26—C27—C28—C29	0.3 (5)
C12—C13—C14—C15	0.9 (5)	C27—C28—C29—C30	−0.3 (5)
C13—C14—C15—C10	0.2 (5)	C28—C29—C30—C25	−0.3 (5)
C11—C10—C15—C14	−1.2 (5)	C26—C25—C30—C29	0.9 (5)
C2—C10—C15—C14	176.8 (3)	C17—C25—C30—C29	−178.1 (3)
